# Quantitative succinylome analysis in the liver of non-alcoholic fatty liver disease rat model

**DOI:** 10.1186/s12953-016-0092-y

**Published:** 2016-02-03

**Authors:** Yang Cheng, Tianlu Hou, Jian Ping, Gaofeng Chen, Jianjie Chen

**Affiliations:** Department of liver disease, Hospital for Infectious Diseases of Pudong New Area, Shanghai, 201299 P. R. China; Shuguang Hospital affiliated to Shanghai University of Traditional Chinese Medicine, Shanghai, 201203 P. R. China

**Keywords:** Non-alcoholic fatty liver disease, Lysine succinylation, Succinylome, Bioinformatics analysis

## Abstract

**Background:**

Non-alcoholic fatty liver disease (NAFLD) is a clinical frequent disease. However, its pathogenesis still needs further study, especially the mechanism at the molecular level. The recent identified novel protein post-translational modification, lysine succinylation was reported involved in diverse metabolism and cellular processes. In this study, we performed the quantitative succinylome analysis in the liver of NAFLD model to elucidate the regulatory role of lysine succinylation in NAFLD progression.

**Methods:**

Firstly, experimental model of NAFLD was induced by carbon tetrachloride injection and supplementary high-lipid and low-protein diet. Then series histochemical and biochemical variables were determined. For the quantitative succinylome analysis, tandem mass tags (TMT)-labeling, highly sensitive immune-affinity purification, liquid chromatography-tandem mass spectrometry techniques were applied. Bioinformatics analysis including gene ontology annotation based classification; Wolfpsort based subcellular prediction; function enrichment; protein-protein interaction network construction and conserved succinylation site motifs extraction were performed to decipher the differentially changed succinylated proteins and sites and *p*-value < 0.05 was selected as threshold.

**Results:**

Totally, 815 succinylation sites on 407 proteins were identified, of which 243 succinylation acetylation sites on 178 proteins showed changed succinylation level with the threshold fold change > 1.5. Theses differentially changed succinylated proteins were involved in diverse metabolism pathways and cellular processes including carbon metabolism, amino acid metabolism, fat acid metabolism, binding and catalyzing, anti-oxidation and xenobiotics metabolism. Besides, these differentially changed succinylated proteins were prominently localized to cytoplasm and mitochondria. Moreover, 8 conserved succinylation site motifs were extracted around the succinylation sites.

**Conclusions:**

Protein succinylation was an extensive post-translation modification in rat. The changed succinylation level in diverse proteins may disturb multiple metabolism pathways and promote non-alcoholic fatty liver disease development. This study provided a basis for further characterization of the pathophysiological role of lysine succinylation in NAFLD progression, which laid a foundation for the innovation of novel NAFLD drugs and therapies.

**Electronic supplementary material:**

The online version of this article (doi:10.1186/s12953-016-0092-y) contains supplementary material, which is available to authorized users.

## Background

Non-alcoholic fatty liver disease (NAFLD) is an insulin resistance and genetic susceptibility related metabolic stress induced liver injury, including non-alcoholic simple fatty liver (NAFL), non-alcoholic steatohepatitis (NASH) and cirrhosis [1]. With the age of onset of obesity and it induced metabolic disorder becoming younger, the injury of NAFLD on liver gets progressively worse. In 2009, investigation on 3175 adults in Shanghai indicated that the rate of NAFLD was 17.29 % [2]. However, the rate ascended to 23.3 % in Shanghai and increased rapidly in the major cities of China in 2012 [3]. NAFLD now is the second most common liver disease after viral hepatitis in China [2, 3]. A well-known two-hit hypothesis has been proposed for the demonstration of the pathogenesis of NAFLD. According to this hypothesis, excess fat accumulated in the liver firstly, then oxidative stress and lipid peroxidation led to hepatic inflammation, fibrosis and a progressive form NASH and to cirrhosis [4]. However, the underlying deep mechanisms are still unclear and approved pharmacological agents are unavailable for NAFLD [4]. Undoubtedly, NAFLD places an enormous economic burden on society and lowers the quality of one’s life greatly. It’s profound to study NAFLD pathogenesis and explore novel cure avenues.

Protein post-translational modifications (PTMs) are important regulatory patterns in a multiple of cellular events [5, 6]. PTMs are defined as covalent processing events that change the properties of a protein by proteolytic cleavage or by adding a modified group to one or more amino acids, thus the activity stage, localization, turnover and interactions with other proteins were changed [7]. Among all the amino acids, lysine is a frequent target to be modified, which can be subjected to a variety of PTMs including methylation, acetylation, biotinylation, ubiquitination, ubiquitin-like modifications, propionylation and butyrylation [8–11]. Increasing novel PTMs were identified recent years. Identifying various potential novel PTMs in diverse species and demonstrating the biological roles of these novel PTMs in organism have been a research hotspot, such as the study of protein propionylation, butyrylation and succinylation [9, 12, 13].

Recent studies indicated that some PTMs were closely connected with liver diseases. Kendrick et al. studied lysine acetylome in mice liver and found multiple hyper-acetylated proteins were presented in the liver of high-fat diet mice [14]. Albitar et al. reported that the ubiquitination of certain proteins was related with chronic liver disease and they developed an ubiquitin proteasome system profiling for the diagnosis of chronic liver disease [15]. However, the role of PTMs, especially some recently identified PTMs such as lysine succinylation and lysine malonylation in liver disease still need further study.

In the present study, we carried out the quantitative analysis of lysine succinylation in the liver of NAFLD rat model. A series of bioinformatics analysis were conducted to explore the molecular mechanisms of NAFLD genesis and progress, where the change of protein succinylation level may be involved. We aim to explore the effects of protein succinylation on the pathogenesis of NAFLD and probe potential diagnostic biomarkers and/or therapeutical targets of NAFLD by antibody based immunoprecipitation affinity enrichment and HPLC-MS/MS based proteome analysis.

## Results

### Morphological changes in NAFLD rat model

**Fig. 1:**
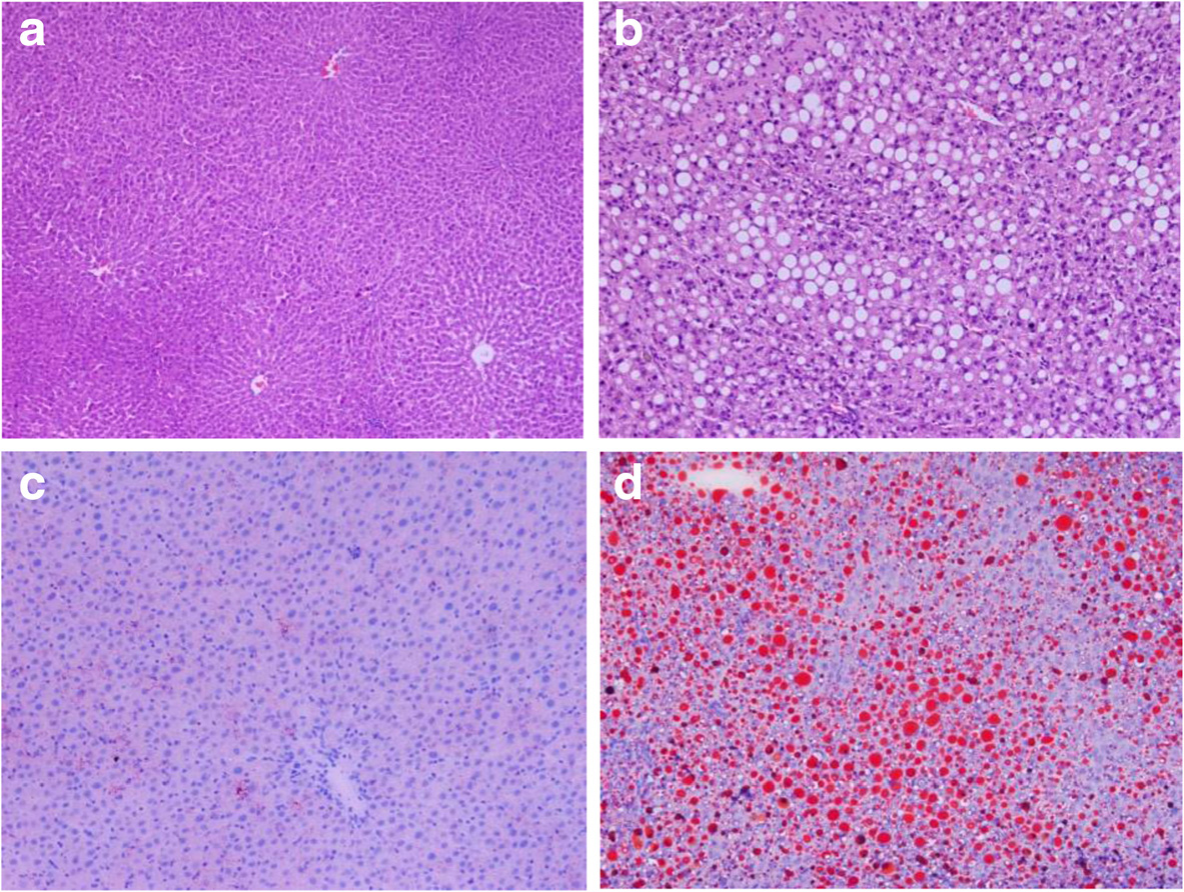
Tissue morphological characteristics in the liver of NAFLD rat model and normal control. HE staining in normal control (**a**) and Non-alcoholic fatty liver disease (**b**); oil red O staining in normal control (**c**) and Non-alcoholic fatty liver disease (**d**)

Initially, hemotoxylin and eosin (HE) staining was applied to observe the liver pathological changes in NAFLD rats. The results showed that, in control group (Fig. 1a), the structure of liver tissue was complete and the structure of sinus hepaticus was clearly observed; Cells in the tissue were with normal morphology and presented radial arrangement with the central vein as the center. In contrast, structures of liver acini hepatis in NAFLD rats were disorganized, and cells in the liver were degenerated with cytoplasm rarefaction and large number of fat vacuoles (Fig. 1b). Further oil red O staining from frozen section indicated that few lipid droplets were observed in the liver tissues of normal rats (Fig. 1c), while lots of fused lipid droplets were presented in the liver tissues of NAFLD rats (Fig. 1d).

### Biochemical changes of NAFLD rat model

As shown in Table 1, the glycerin trimyristate (TG) content in the liver of NAFLD model was significantly higher than that in the liver of normal control. What’s worse, the serum alanine aminotransferase (AST), alanine aminotransferase (ALT) and glutamyltranspetidase (GGT) activities in NAFLD rats also ascended significantly compared with the control group, which indicated that liver dysfunction may have occurred in the NAFLD model. Antioxidant index determination results indicated that the activity of superoxide dismutase (SOD) and the content of glutathione (GSH) declined significantly in the NAFLD group while the content of malonaldehyde (MDA) increased significantly compared with the control group, implying the liver of NAFLD rats suffered severe oxidative damage. Overall, we constructed a NAFLD rat model successfully.

**Table 1:** Biochemical variable determination

	Normal control group	NAFLD group
Liver tissue TG content (mg/g)	12.4 ± 1.5	75.6 ± 13.4
Serum ALT activity (U/L)	47 ± 9	172 ± 37
Serum AST activity (U/L)	126 ± 11	295 ± 43
Serum GGT activity (U/L)	6 ± 1.5	58 ± 5.3
Liver tissue SOD activity (U/mg protein)	214 ± 35	106 ± 23
Liver tissue MDA content (mg/g protein)	19 ± 8	75 ± 23
Liver tissue GSH content (mg/g protein)	21 ± 3.7	9 ± 2.3

Notes: Each value is the mean ± SE (*n* = 10) and *p* value less than 0.01 was regarded as statistic significant

### General characterization of the quantitative succinylome in rat liver tissues

A total of 815 succinylation sites on 407 proteins were identified with two technical repeat experiments (686 succinylation sites in the first experiment and 692 in the second one, among which 563 sites were identified in both experiments). With the threshold fold change > 1.5, 243 succinylation sites corresponding to 178 proteins showed different succinylation level in our two technical repeat experiments (180 up-regulated succinylated sites on 132 proteins, 94 down-regulated succinylated sites on 46 proteins, NAFLD group compared with normal control group). All the identified succinylated sites and corresponding proteins were summarized in Additional file 1.

**Fig. 2:**
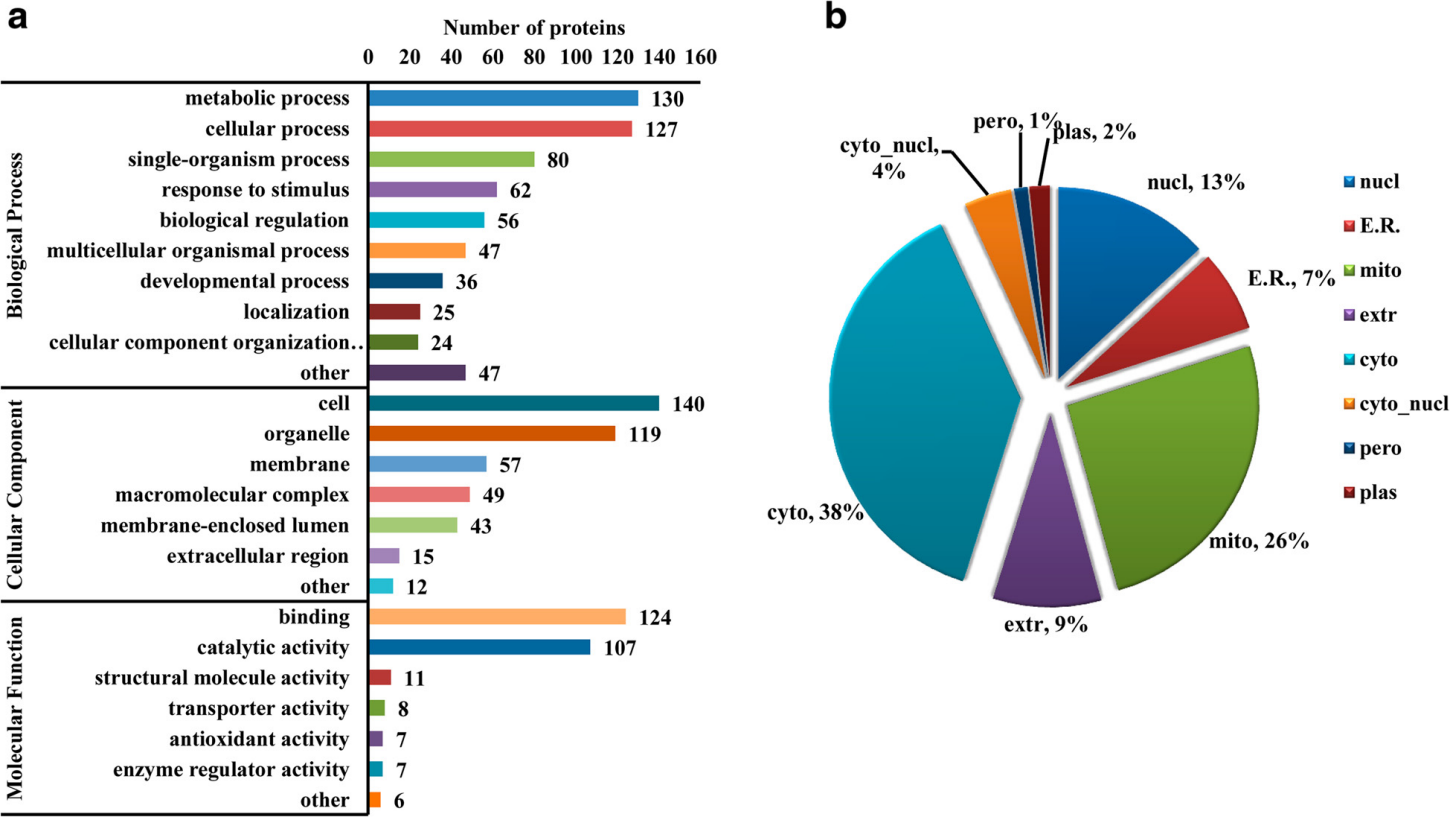
GO annotation based function classification and Wolfpsort based subcellular localization prediction. Function distribution of the identified differentially changed succinylated protein from GO analysis (**a**) and subcellular localization prediction from Wolfpsort (**b**). With the threshold change fold > 1.5, all the 178 differentially changed succinylated proteins (The proteins with down-regulated or up-redulated succinylation level in the Additional file 1, NAFLD/Control Ratio > 1.5 or <0.67) were used for these analysis

GO annotation based classification and Wolfpsort based subcellular prediction analysis were conducted to investigate the nature of these differentially changed succinylated proteins (Fig. 2). The classification result on category of biological process showed that metabolic process and cellular process related proteins were the two largest groups suffered changed succinylation level. Molecular function analysis indicated the major differentially changed succinylated proteins were binding and catalytic activity related, whose percentage was 45.9 % and 39.6 %, respectively. Cellular component based classification analysis found cell and organelle related proteins were the main proteins with changed succinylation level while other cellular component related proteins were relatively less (Fig. 2a).

Subcellular location prediction result (Fig. 2b) showed the majority of these differentially changed succinylated proteins were localized to cytoplasm (38 %) and mitochondria (26 %), followed by nucleus (13.1 %), extracellular (6.9), endoplasmic reticulum (ER, 6.9 %), cytonucl (4.0 %), plasm (1.7 %) and peroxisome (1.1 %).

### Enrichment analysis of the differentially changed succinylated proteins

**Fig. 3:**
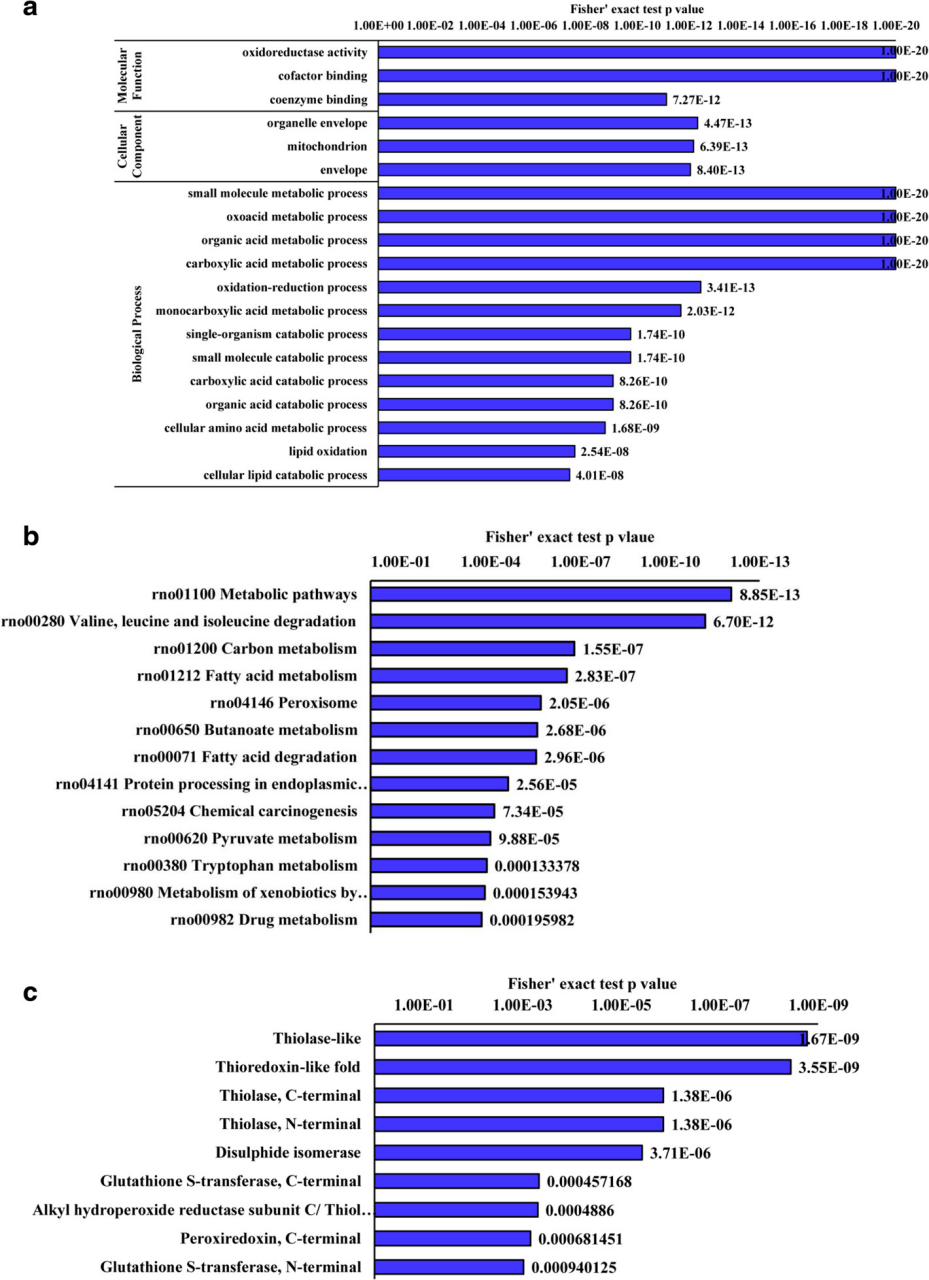
Enrichment analysis of the differentially changed succinylated proteins. GO annotation based enrichment analysis (**a**), KEGG pathway enrichment analysis (**b**) and domain enrichment analysis (**c**). With the threshold change fold > 1.5, all the 178 differentially changed succinylated proteins (The proteins with down-regulated or up-redulated succinylation level in the Additional file 1, NAFLD/Control Ratio > 1.5 or <0.67) were used for these analysis. The GO enrichment analysis were performed on the ontology of molecular function, cellular component and biological process. DAVID as selected as the tool and the adjusted p-value less than 0.05 was chosen as cut-off criterion

As shown in (Fig. 3a), on molecular functions ontology, the top three enriched GO terms were oxidoreductase activity, cofactor binding and coenzyme binding. The markedly enriched cellular components were organelle envelope, mitochondrion and envelope (Fig. 3a), signifying mitochondrion and/or other enveloped structures may be the place where the proteins were differentially succinylatation modified with a relatively higher frequency. On the ontology of biological processes, the majority of these markedly enriched terms were concerned with the metabolism of small molecules, such as oxoacid, organic acid, carboxylic acid, amino acid and lipid (Fig. 3a).

In the KEGG pathway enrichment analysis, the top three enriched pathways were metabolic pathways, valine, leucine and isoleucine degradation and carbon metabolism. Besides, many other amino acid metabolism and fat acid metabolism related pathways were also enriched (Fig. 3b)

Pfam domain analysis revealed that the top two significantly enriched terms were thiolase-like and thioredoxin-like fold. In addition, thiolase C-terminal and thiolase, N-terminal domain was also significantly enriched with high confidence. What’s noticeable was that all the significantly enriched protein domains contained active group with sulfur or sulphydryl (Fig. 3c). We infer the activity of some sulfur /sulphydryl containing proteins/enzymes may be changed by succinylation or dysuccinylation and these changes may be involved in the progression of NAFLD.

### Protein protein interaction (PPI) network analysis

**Fig. 4:**
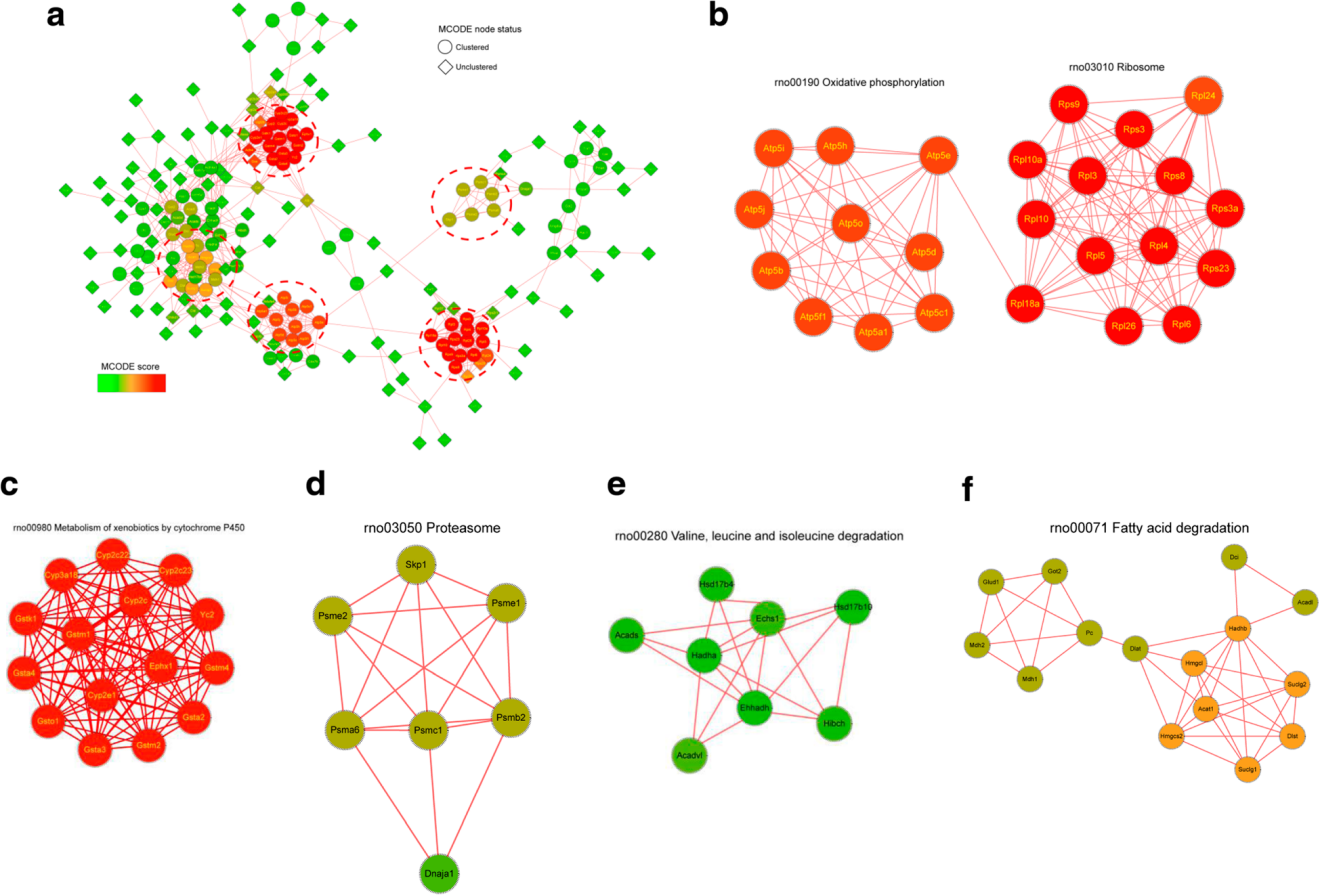
The protein-protein interaction network analysis. **a** the whole interaction network; **b**-**f**, the extracted significantly enriched function models. With the threshold change fold > 1.5, all the 178 differentially changed succinylated proteins (The proteins with down-regulated or up-redulated succinylation level in the Additional file 1, NAFLD/Control Ratio > 1.5 or <0.67) were used for these analysis. STRING database was used to annotate functional interactions of all the identified differentially succinylated proteins. The MCODE plug-in toolkit was used to identify highly connected clusters and the interaction network was visualized by Cytoscape software (version 3.0.1)

To illuminate the mechanism of protein succinylation mediated NAFLD genesis and development, we constructed the PPI network for all the succinylated proteins with STRING database and Cytoscape software (Fig. 4). With MCODE plug-in toolkit, a total of 5 highly enriched interaction clusters were obtained, which were related with metabolism of xenobiotics by cytochrome P450, oxidative phosphotylation and ribosome, proteasome, fatty acid degradation and valine, leucine and isoleucine degradation.

### Characterization of succinylated lysine sites in rat liver

**Fig. 5:**
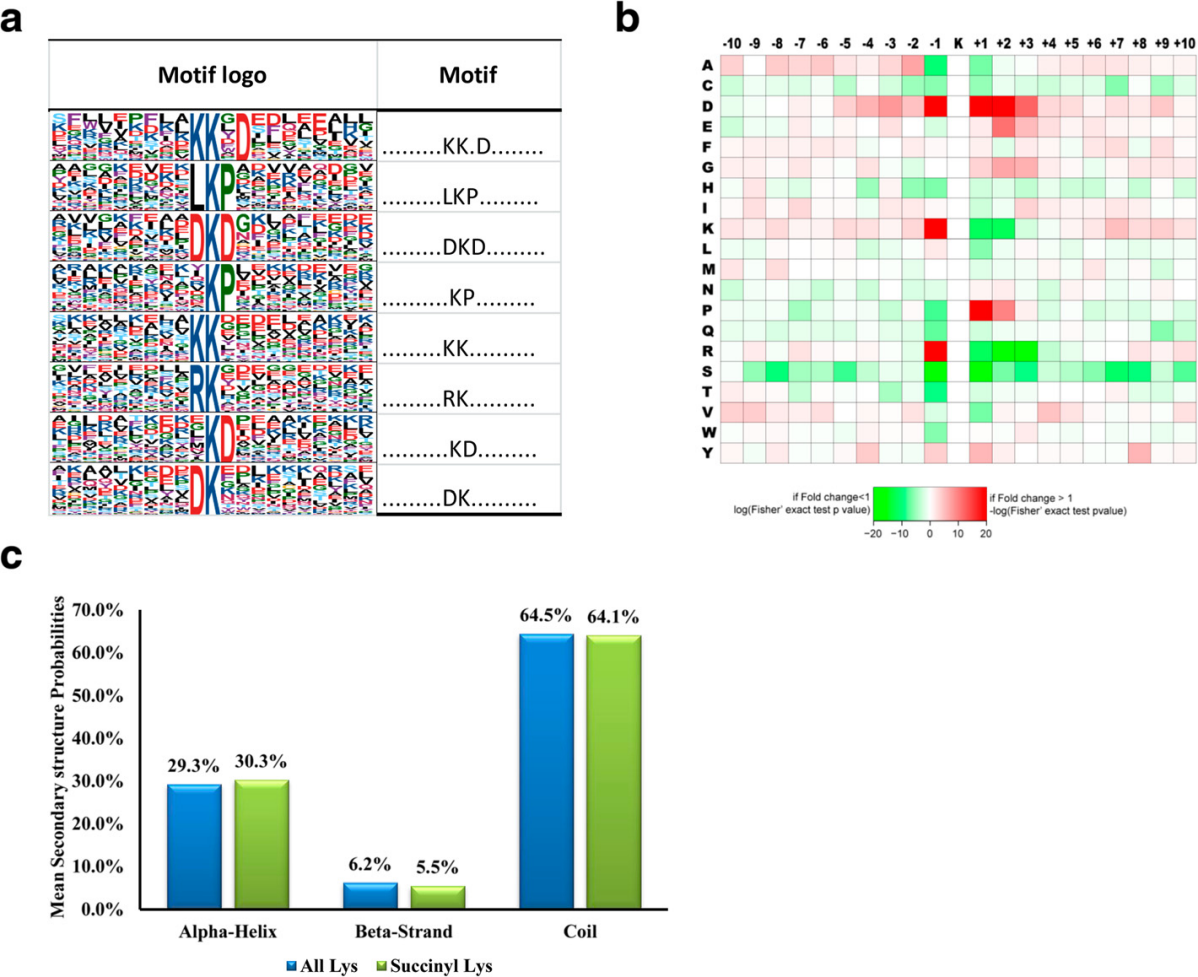
Properties of the succinylated peptides. **a** Succinylation motifs and conservation of succinylation sites. **b** Heat map of the amino acid compositions of the succinylated site. **c** Distribution of succinylated lysines and all lysines in protein secondary structures. All the identified 815 succinylation sites (Additional file 1) were used for the analysis. Software motif-x was used to analysis the model of sequences constituted with amino acids in specific positions of succinyl-21-mers (10 amino acids upstream and downstream of the site). The local secondary structures of succinylated proteins were predicted by NetSurfP. *P*-value < 0.05 was considered significant for these analysis

As shown in Fig. 5a, a total of 8 definitively conserved succinylation site motifs were defined, namely K^su^ K*D, LK^su^P, DK^su^D, K^su^P, KK^su^, RK^su^, K^su^D, DK^su^ (K^su^ represents the succinylated lysine and * represents a random amino acid residue). To determine whether there are specific amino acids adjacent to succinylated lysines, we examined the amino acid sequences flanking succinylation sites by heat'map (Fig. 5b). Aspartic acid (D), lysine (K) and arginine (R) were overrepresented in the −1 position of succinylation sites. D was also appeared in the +1 to +3 positions with high frequency while K and R appeared in these positions with very low frequency. Besides, a special amino acid, proline (P) appeared in the +1 position of succinylation sites frequently but seldom appeared in the −1 position. In addition, it seemed that Serine (S) was unwelcomed surrounding the succinylation sites as its frequency of occurrence was obviously lower than other amino acids in both upstream and downstream of succinylation sites.

Secondary structure analysis showed that the succinylation sites distribution was about 64.1 % in coil, 30.3 % in helix, and 5.5 % in beta-strand (Fig. 5c).

## Discussion

Lysine succinylation is a newly identified PTM and impacts diverse metabolic pathways [16]. Recent protein succinylome studies in mouse liver and mouse embryonic fibroblasts (MEFs) showed that proteins succinylation level changes participated in fat acid metabolism related pathways such as fatty acid β-oxidation and long-chain fatty acid transportation [16–18]. It was well known that various fatty liver diseases including nonalcoholic simple fatty liver and alcoholic fatty liver were highly related with liver fatty acid metabolism dysfunction. Thus we investigated the quantitative protein succinylome in NAFLD rat model, with the purpose of exploring the possible roles of lysine succinylation in NAFLD progression.

Firstly, we constructed a classical NAFLD rat model according to previous report [19] and a series of histological, physiological and biochemical variables were detected. High fat and low protein diet induced over accumulation of TG, disturbance of biochemical variables and serious oxidative damage (Fig. 1 and Table 1), which was consistent with previous report [19].

Lysine succinylome analysis identified 815 succinylation sites on 407 proteins in the liver tissues of NAFLD rat model and normal control. Park et al. identified 1,675 sites from 436 proteins in liver tissues of Sirt5 knockout mouse [18]. The huge difference of the volume of succinylated sites may be related with the knockout of Sirt5 as Sirt5 can remove succinyl moieties from target lysine and acts as a desuccinylase role [20]. Quantification analysis indicated that approximately two thirds proteins showed elevated succinylation level in NAFLD group compared with control group. These hyper-succinylated proteins may be involved in the genesis and development of NAFLD.

The classification result (Fig. 2a) implied that changed lysine succinylation level may have influenced many metabolic processes and cellular processes in NAFLD rat liver. GO and KEGG pathway enrichment analysis (Fig. 3a and b) further supported this hypothesis as many small molecules (oxoacid, organic acid, carboxylic acid, amino acid, fatty acid and lipid etc.) metabolism processes and pathways were significantly enriched. Previous studies have well demonstrated the role of abnormal metabolism of aboveing metabolites in NAFAD pathogenesis [21–23]. A recent study on mouse liver lysine succinylome also revealed many lysine-succinylated proteins were predominantly involved in fatty acid metabolism, amino acid degradation and the tricarboxylic acid cycle [16]. Here we infer the succinylation level alteration (preferentially hyper-succinylation) on these molecules metabolism related proteins and/or enzymoses may be a potential promoting mechanism of NAFLD progression.

High proportion binding and catalytic related proteins (Fig. 2a) and significantly enriched cofactor binding and coenzyme binding terms (Fig. 3a) imply changed succinylation level may perturb the normal catalyzing or binding function of target proteins/enzymes. These proteins/enzymes were succinylation modified, thus their space structure, charge states and stability may be altered and they can’t bind to their coenzymes or cofactors successfully, which further disturbed their binding ability and catalytic activity. Succinylation disturbed substrate binding and enzyme catalytic activity had been reported in isocitrate dehydrogenase [24]. What’s worse, succinylation adds a bigger structural moiety than acetylation or methylation and it is likely to lead to more significant changes in protein structure [24], which may change the protein binding or catalyzing activity seriously.

What’s noticeable was that oxidoreductase activity term was the top significant enriched term in molecular function enrichment analysis; functional classification analysis also identified 7 antioxidant ability related proteins. Correspondingly, we observed aggravated reactive oxygen species (ROS) stress and deteriorated anti-oxidation enzyme system in NAFLD by biochemical variables determination. We guess the changes of succinylation level on antioxidant enzymes may be another important event in NAFLD development. The antioxidant related proteins with changed succinylation level might be potential targets for NAFLD treatment.

Subcellular location prediction result indicated cytoplasm located differentially changed succinylated proteins accounted for the largest part, and then was the mitochondria located proteins. In a recent study, cellular compartment analysis of all the lysine-succinylated proteins identified from Sirt5 knockout (KO) liver tissue and mouse embryonic fibroblasts (MEFs) found mitochondria located succinylated proteins accounted for the largest part while cytoplasm located proteins was the second most proteins [18]. Another succinylome study in mouse liver tissue also observed the similar trends [16]. The difference of subcellular distribution may be related with the different experiment materials or experimental treatments. For example, Sirt5 is an inhibitor of lysine succinylation in mitochondria [18], Sirt5 knockout naturally induced more protein succinylation modification in mitochondria.

PPIs are crucial for most cellular processes [25]. Various PTMs can change the interactions among protein, thus metabolism processes and cellular processes were affected [7]. In the obtained 5 significantly enriched function modules, we found function module metabolism of xenobiotics by cytochrome P450, fatty acid degradation and valine, leucine and isoleucine degradation were also enriched in the preceding GO and KEGG enrichment analysis, which implied their important roles in NAFLD development.

The score of interaction clusters metabolism of xenobiotics by cytochrome P450, oxidative phosphotylation and ribosome were very high, signifying alteration of succinylation level on these proteins may contribute to the development of NAFLD. In the metabolism of xenobiotics by cytochrome P450 function module, most of these interacted proteins belonged to the Cytochrome P450 (CYP450) family or Glutathione S-transferase (GST) family. These two families participated in the metabolism of various metabolites together, especially the metabolism of secondary metabolism such as steroids, fatty acids, xenobiotics and so on [26–28]. Lysine succinylation on the members of CYP450 family and GST family may disturb various secondary metabolism and induced NAFAD. This is consistent with the aforementioned classification and enrichment analysis as various molecular metabolism related terms were significantly enriched. The major proteins in oxidative phosphotylation and ribosome cluster were subunits of mitochondrial F0F1 ATP synthase and ribosome, which imply succinylation on F0F1 ATP synthase and ribosome were important events in NAFAD development. The energy production and protein synthesis was likely to be interfered by increased lysine succinylation modification in the liver of NAFLD model.

In the motif analysis, we identified 8 definitively conserved succinylation site motifs. Previous motif and flanking amino acid sequence analysis in SIRT5 knockout mouse showed that positively charged amino acids (lysine or arginine) were strongly excluded from positions −1 and +1 of the succinylation logo [17, 18]. Our study indicated lysine and arginine were rare represented in +1 position while over represented in the −1 position of succinylation sites. Furthermore, the negatively charged amino acid aspartic acid was highly represented in the positions near succinylation sites (−1, +1, +2, +3). In the secondary structure analysis, we observed a slight bias for succinylation to occur in alpha helix regions and a slight bias against beta-strand regions and coil regions, which was similar with previous finding that succinylation sites have moderate local structural preferences for helical regions and a moderate bias against strand and coiled regions in mouse [16].

## Conclusions

In conclusion, we performed the quantitative succinylome analysis in the liver of NAFLD rat model. A total of 815 succinylation sites from 407 proteins were identified, of which 243 succinylation sites corresponding to 178 proteins had shown changed succinylation level with the threshold fold change > 1.5. Bioinformatics analysis indicated that these differentially changed succinylated proteins participated in various metabolism processes and cellular processes including but not defining to carbon metabolism, amino acid metabolism, fat acid metabolism, binding and catalyzing, anti-oxidation and xenobiotics metabolism. Alteration of succinylation level on these metabolism and cellular processes related proteins may have changed many normal metabolism pathways and promoted NAFLD progression. These proteins with differential succinylation level could be the potential diagnose biomarkers/therapy targets for NAFLD treatment. Besides, the hyper-succinylated proteins were mainly localized to cytoplasm and mitochondria. Motif analysis obtained 8 conserved succinylation site motifs, namely K^su^ K*D, LK^su^P, DK^su^D, K^su^P, KK^su^, RK^su^, K^su^D and DK^su^. This is the first quantitative succinylome analysis in the liver tissues of NAFLD rat model, which confer a novel perspective for the elucidation of the mechanism underling NAFLD genesis and development as well as the innovation of new drugs and therapy avenues to cure NAFLD.

## Materials and Methods

### Experiment design

The purpose of this study was to perform the quantitative lysine succinylome analysis in the NAFLD rat model. Firstly we established the rat model according to previous report. After different treatments, the liver tissue morphological observation and biochemical variables measurement were conducted in both NAFLD model group and normal control group. Then we performed the quantitative succinylation analysis in the liver of NAFLD rat model by using tandem mass tags (TMT)-labeling, antibody-based succinylated peptides affinity enrichment and nano LC-MS/MS techniques. Lastly, bioinformatics analysis were carried out for the systematic interpretation of the identified lysine succinylated sites and succinylated proteins. Two technical repeats were performed for the succinylome analysis.

### Regents

All reagents unless otherwise stated were purchased from Sigma (St. Louis, America). Alanine aminotransferase (ALT), aspartate aminotransferase (AST), glutamyltranspetidase (GGT), glycerin trimyristate (TG), superoxide dismutase (SOD), reduced glutathione (GSH) and malonaldehyde (MDA) assay kit were purchased from Nanjing Jiancheng Bioengineering Institute (Nanjing, China). Anti-succinyl lysine antibody agarose beads were purchased from PTM Biolabs (Hangzhou, China).

### Experimental animal model

The detailed description of the model establishment and diet for rats were given in the Additional file 2. Twenty male Wistar rats with SPF grades, weight range 170 ± 10 g were used in this study and the rat model was established according to Yao et al. [19]. The animal studies and protocols were approved by the Experimental Animal Ethics Committee of Hospital for Infectious Diseases of Pudong New Area.

### Liver tissue preparation

Four weeks after treatment, all rats were fasted but normally supplied with water for 24 h. Then rats were performed peritoneal injection with 3 % pentobarbital sodium according to injection dose to rat weight of 2 ml/Kg. Abdominal cavity of anesthetic rat was incised, and then inferior vena cava blooding sampling was performed with 10 ml syringe. After standing for 3 h at 4 °C, blood samples were centrifuged at 5000 x g for 15 min. Supernatant was saved at −80 °C for future use. Liver was incised and two segments (1.0 cm x 1.0 cm x 0.3 cm) were cut from right lobe of the liver. Two liver segments were fixed in 10 % neutral formalin and embedded in opt-imum cutting temperature compound (OCT). The remained liver samples were saved at −80 °C for future use.

### Morphologic observation

To observe liver tissues morphologic changes, the HE staining [29] and red O staining [30] of liver segments was performed according to previous description.

### Biochemical Variable Determination

Activities of AST, ALT and GGT in serum, and TG content, MDA content, GSH content and SOD activity in liver tissues from experimental rats were determined according to the instructions provided by the assay kit [31].

### Proteomic analysis

#### Sample preparation

The protein extraction of the rat liver tissues was performed according to previous report [32] and digested by trypsin (Promega) with the second digestion method to ensure thorough digestion [33].

Briefly, the liver tissues were first grinded by liquid nitrogen and the powder was transferred to 5 mL centrifuge tube and precipitated with cold 10 % TCA/acetone supplemented with 50 mM DTT, 0.1 % Protease Inhibitor Cocktail Set IV and HDACinhibitor (50 mM sodium butyrate, 30 mM nicotinamide, and 3 μM Trichostatin A) for 2 h at −20 °C. After centrifugation at 20,000 g at 4 °C for 10 min, the resulting precipitate was washed with cold acetone for three times and air dried. Then the precipitate was re-suspended in lysis buffer (8 M urea, 2 mM EDTA, 10 mM DTT) and sonicated three times on ice using a high intensity ultrasonic processor (Scientz) and the remaining debris was removed by centrifugation.

The supernatant was reduced with 10 mM DTT for 1 h at 56 °C and alkylated with 55 mM IAA for 45 min at room temperature in darkness. Afterwards, the protein was precipitated with 3 volumes of pre-chilled acetone for 30 min at −20 °C. After centrifugation, the pellet was then dissolved in 0.5 M TEAB and sonicated for 5 min. Repeat the centrifugation step and collect the supernatant. Protein content in the supernatant was determined with 2-D Quant kit according to the manufacturer’s instructions. The protein was then digested with trypsin (Promega) at an enzyme-to-substrate ratio of 1:50 for 12 h at 37 °C. To insure complete digestion and improve protein identification and characterization, additional trypsin at an enzyme-to-substrate ratio of 1:100 was added, and the mixture was incubated for an additional 4 h. After trypsin digestion, peptide was desalted by Strata X C18 SPE column (Phenomenex) and vacuum-dried. Then peptides were labeled with a 2-plex TMT kit with the protocol of the manufacture.

#### Enrichment of succinylated lysine peptides

Enrichment of lysine succinylated peptides was implemented by immunoprecipitation according to previous report [34] and the anti-succinyl lysine antibody agarose conjugated beads (PTM Biolab) was used with a ratio of 15 μL beads/mg protein.

Briefly, 5 mg tryptic peptides was re-dissolved in NETN buffer NETN buffer (100 mM NaCl, 1 mM EDTA, 50 mM Tris–HCl, 0.5 % NP-40, pH 8.0) and incubated with anti-succinyl lysine antibody agarose conjugated beads (PTM Biolab) in a ratio of 15 μL beads/mg protein at 4 °C overnight with gentle end-to-end rotation. After incubation, the beads were washed four times with NETN buffer and twice with purified water. The bound peptides were eluted with 1 % trifluoroacetic acid (TFA) and dried under a vacuum. The eluted peptides were cleaned with C18 ZipTips (Millipore) in accordance with the manufacturer's instructions followed by HPLC/MS/MS analysis.

#### LC-MS/MS

LC-MS/MS was performed according to previous report [13] and the detailed process and parameters were shown in Additional file 2.

#### Database Search

All the detailed parameters were shown in the Supporting Information (Additional file 2). The protein and succinylation sites were identified using MaxQuant software (Version. 1.0.13.13) and Andromeda search 172 engine (Version 1.4.1.2). Tandem mass spectra were searched against *Uniprot_Rat* database with reverse decoy database.

### Succinylation Quantification

The quantification of the succinylated peptides and proteins were calculated according to the TMT reporter ion intensities with COMPASS v1.2.1.0 software [35]. All peptides with same succinylation patterns were grouped together and their reporter ion intensities were summed. The quantitative ratios were weighted and normalized by the median ratio. The manufacturer’s recommended isotope correction factors were used. Based on relative quantification and statistical analysis, 1.5-fold change was set as threshold for differentially changed succinylated proteins.

### Bioinformatics Analysis

The databases and softwares for bioinformatics analysis were shown in Additional file 2. When performing the bioinformatics analysis, *p*-value < 0.05 was considered significant.

### Statistical methods

Data were processed by using SPSS 17.0. Measurement data were indicated as mean ± SEM. Comparisons between groups were tested by One -Way ANOVA analysis and statistical difference was determined when *P* < 0.05.

## Additional files

Additional file 1:
**The summary of all the identified succinylation sites and corresponding proteins.** (XLSX 71 kb) Additional file 2:
**The detailed description of the experiment methods, including rat model establishment, mass spectrometric analysis procedures and parameters, bioinformatics analysis softwares, websites.** (DOCX 18 kb)

## Abbreviations

NAFLD: Non-alcoholic fatty liver disease
PTM: Post-translational modification
PPI: Protein-protein interaction
GO: Gene Ontology
KEGG: Kyoto Encyclopedia of Genes and Genomes
HE: Hemotoxylin and eosin
CCL4: Analytical grade carbon tetrachloride
ALT: Alanine aminotransferase
AST: Aspartate aminotransferase
GGT: Glutamyltranspetidase
TG: Glycerin trimyristate
SOD: Superoxide dismutase
GSH: Reduced glutathione
MDA: Malonaldehyde
ROS: Reactive oxidative stress
LC-MS/MS: Liquid chromatography-mass spectrometry/ mass spectrometry
