# Supplementary material for: Quantitative succinylome analysis in the liver of non-alcoholic fatty liver disease rat model
Source: Proteome Sci. 2016 Feb 3;14:3. doi: 10.1186/s12953-016-0092-y (PMC4739109; doi:10.1186/s12953-016-0092-y)
Supplement: Additional file 2: — The detailed description of the experiment methods, including rat model establishment, mass spectrometric analysis procedures and parameters, bioinformatics analysis softwares, websites. (DOCX 18 kb) [file 12953_2016_92_MOESM2_ESM.docx]

**The detailed description of experiment methods**

**Rat model establishment**

One week after adaptive feeding, twenty rats were randomly divided as experimental group (10 rats) and control group (10 rats). Rats in experimental group were performed peritoneal injection with 40% CCl_4_ in olive oil according to injection dose to rat weight of 1 ml/Kg, twice injection per week and 8 injections in total. Rats in control group were performed peritoneal injection with same volume of physiological saline with same frequency. Rats in experimental group were fed with high fat and low protein diet (79.5% corn flour, 20% lard, 0.5% cholesterol) in the 1st and 2nd week, and sequentially fed with pure corn flour diet in the 3rd and 4th week. Rats in control group were fed with regular diet at all time. Water was freely supplied to rats in two groups at all time. All diets were offered by experimental animal facility of Shanghai University of Traditional Chinese Medicine.

**HPLC-MS/MS**

The peptides were re-suspended in buffer A (0.1% FA, 2% ACN) and centrifuged at 20000g for 2min. The supernatant was transferred into sample tube and a reversed-phase column (360 μm OD × 75 μm ID) packed in-house with 3-μm C18 beads (Reprosil-Pur C18-AQ, Dr. Maisch) and eluted with a linear gradient of 5–35% buffer B (0.1% FA in 98% ACN) for 30 min and 35-80% solvent B for 10 min at a constant flow rate of 300 nl/min on an EASY-nLC 1000 UPLC system (Thermo Scientific). The resulting peptides were analyzed by Q ExactiveTM Plus hybrid quadrupole-Orbitrap mass spectrometer (Thermo Fisher Scientific).

The peptides were subjected to a NanoSpray Ionization (NSI) source followed by MS/MS in Q Exactive (Thermo Scientific) coupled online to the UPLC. Intact peptides were detected in the Orbitrap at a resolution of 70,000. Peptides were selected for MS/MS using 25% Normalized Collision Energy (NCE) with 4% stepped NCE; ion fragments were detected in the Orbitrap at a resolution of 17,500. A data-dependent procedure that alternated between one MS scan followed by 20 MS/MS scans was applied for the top 20 precursor ions above a threshold ion count of 3E4 in the MS survey scan with 15.0s dynamic exclusion. The electrospray voltage applied was 1.8 kV. Automatic gain control (AGC) was used to prevent overfilling of the Orbitrap; 2E5 ions were accumulated for generation of MS/MS spectra. For MS scans, the m/z scan range was 350 to 1600 Da. The fixed first mass was set at 100 m/z for TMT quantification.

**Database Search parameters**

Trypsin/P was specified as cleavage enzyme allowing up to 3 missing cleavages, 4 modifications per peptide and 5 charges. Mass error was set to 10 ppm for precursor ions and 0.02 Da for fragment ions. Carbamidomethylation on Cys was specified as fixed modification, oxidation on Met and succinylation on Lys were specified as variable modifications. Reporter ion was set as 2-plex TMT for quantification. False discovery rate (FDR) thresholds for protein, peptide and modification site were specified at 1%. Minimum peptide length was set at 6. Lysine succinylation sites identified with a localization probability of less than 0.75 from reverse or contaminant protein sequences were removed.

**Bioinformatic analysis**

Protein classification of the different expressed proteins on succinylation level was based on the Gene Ontology (GO) database annotation and subcellular location prediction was performed through Wolfpsort. The tool for enrichment analysis was DAVID and the adjusted p-value less than 0.05 were chosen as cut-off criterion. For protein-proteins interaction analysis, the Search Tool for the Retrieval of Interacting Genes/Proteins (STRING) database and Cytoscape software (version 3.0.1) were used. Software motif-x was used to analysis the model of sequences constituted with amino acids in specific positions of succinyl-21-mers. The local secondary structures of Kac proteins were predicted by NetSurfP.
